# Supplementary material for: Allosteric regulation and crystallographic fragment screening of SARS-CoV-2 NSP15 endoribonuclease
Source: Nucleic Acids Res. 2023 Apr 28;51(10):5255–70. doi: 10.1093/nar/gkad314 (PMC10250223; doi:10.1093/nar/gkad314)
Supplement: gkad314_Supplemental_Files [file gkad314_supplemental_files.zip › Supplementary datasets.zip!/134/Refine_9/01-BUSTER/Cycle-3/shell.01/R_Rfree.html]

# Rxpct factors in resolution bins at cycle 0


|  |  |  |  |  |  |  |  |  |
| --- | --- | --- | --- | --- | --- | --- | --- | --- |
|  | **<d> (Å)**  Rxpct\_Work | Rxpct\_Free | Rxpct\_All | |  |  |  |  |
|  | 15.4464 | 0.3475 | 0.4455 | 0.3512 |  |
|  | 8.9180 | 0.2293 | 0.2524 | 0.2307 |  |
|  | 6.9078 | 0.2263 | 0.2539 | 0.2278 |  |
|  | 5.8382 | 0.2303 | 0.2979 | 0.2326 |  |
|  | 5.1488 | 0.1847 | 0.2074 | 0.1859 |  |
|  | 4.6573 | 0.1645 | 0.1975 | 0.1663 |  |
|  | 4.2841 | 0.1670 | 0.1934 | 0.1685 |  |
|  | 3.9882 | 0.1634 | 0.2178 | 0.1659 |  |
|  | 3.7463 | 0.1708 | 0.2264 | 0.1734 |  |
|  | 3.5436 | 0.1706 | 0.2094 | 0.1727 |  |
|  | 3.3707 | 0.1764 | 0.2125 | 0.1784 |  |
|  | 3.2208 | 0.1849 | 0.2189 | 0.1868 |  |
|  | 3.0893 | 0.1945 | 0.2207 | 0.1957 |  |
|  | 2.9727 | 0.1950 | 0.2089 | 0.1956 |  |
|  | 2.8683 | 0.1979 | 0.2042 | 0.1982 |  |
|  | 2.7743 | 0.1978 | 0.2387 | 0.1997 |  |
|  | 2.6889 | 0.1962 | 0.1984 | 0.1963 |  |
|  | 2.6109 | 0.2035 | 0.2197 | 0.2045 |  |
|  | 2.5394 | 0.2167 | 0.2239 | 0.2171 |  |
|  | 2.4734 | 0.2354 | 0.2536 | 0.2364 |  |
|  | **Overall** | 0.1957 | 0.2276 | 0.1974 |  |

# Rxpct factors in resolution bins at cycle 1


|  |  |  |  |  |  |  |  |  |
| --- | --- | --- | --- | --- | --- | --- | --- | --- |
|  | **<d> (Å)**  Rxpct\_Work | Rxpct\_Free | Rxpct\_All | |  |  |  |  |
|  | 15.4464 | 0.3495 | 0.4476 | 0.3532 |  |
|  | 8.9180 | 0.2299 | 0.2530 | 0.2313 |  |
|  | 6.9078 | 0.2263 | 0.2553 | 0.2280 |  |
|  | 5.8382 | 0.2295 | 0.2953 | 0.2318 |  |
|  | 5.1488 | 0.1841 | 0.2063 | 0.1853 |  |
|  | 4.6573 | 0.1634 | 0.1985 | 0.1654 |  |
|  | 4.2841 | 0.1665 | 0.1918 | 0.1679 |  |
|  | 3.9882 | 0.1624 | 0.2198 | 0.1651 |  |
|  | 3.7463 | 0.1708 | 0.2261 | 0.1734 |  |
|  | 3.5436 | 0.1712 | 0.2094 | 0.1732 |  |
|  | 3.3707 | 0.1765 | 0.2152 | 0.1786 |  |
|  | 3.2208 | 0.1854 | 0.2176 | 0.1871 |  |
|  | 3.0893 | 0.1946 | 0.2193 | 0.1958 |  |
|  | 2.9727 | 0.1953 | 0.2084 | 0.1959 |  |
|  | 2.8683 | 0.1977 | 0.2042 | 0.1981 |  |
|  | 2.7743 | 0.1973 | 0.2382 | 0.1992 |  |
|  | 2.6889 | 0.1957 | 0.2010 | 0.1960 |  |
|  | 2.6109 | 0.2038 | 0.2192 | 0.2047 |  |
|  | 2.5394 | 0.2160 | 0.2232 | 0.2164 |  |
|  | 2.4734 | 0.2343 | 0.2500 | 0.2352 |  |
|  | **Overall** | 0.1956 | 0.2277 | 0.1973 |  |

# Rxpct factors in resolution bins at cycle 2


|  |  |  |  |  |  |  |  |  |
| --- | --- | --- | --- | --- | --- | --- | --- | --- |
|  | **<d> (Å)**  Rxpct\_Work | Rxpct\_Free | Rxpct\_All | |  |  |  |  |
|  | 15.4464 | 0.3504 | 0.4492 | 0.3541 |  |
|  | 8.9180 | 0.2295 | 0.2517 | 0.2309 |  |
|  | 6.9078 | 0.2211 | 0.2575 | 0.2232 |  |
|  | 5.8382 | 0.2255 | 0.2934 | 0.2279 |  |
|  | 5.1488 | 0.1812 | 0.2168 | 0.1831 |  |
|  | 4.6573 | 0.1638 | 0.2026 | 0.1659 |  |
|  | 4.2841 | 0.1678 | 0.1969 | 0.1694 |  |
|  | 3.9882 | 0.1656 | 0.2275 | 0.1685 |  |
|  | 3.7463 | 0.1742 | 0.2336 | 0.1769 |  |
|  | 3.5436 | 0.1744 | 0.2198 | 0.1768 |  |
|  | 3.3707 | 0.1796 | 0.2156 | 0.1816 |  |
|  | 3.2208 | 0.1865 | 0.2216 | 0.1884 |  |
|  | 3.0893 | 0.1950 | 0.2273 | 0.1965 |  |
|  | 2.9727 | 0.1940 | 0.2052 | 0.1944 |  |
|  | 2.8683 | 0.1972 | 0.1982 | 0.1972 |  |
|  | 2.7743 | 0.1964 | 0.2318 | 0.1981 |  |
|  | 2.6889 | 0.1938 | 0.1920 | 0.1937 |  |
|  | 2.6109 | 0.1999 | 0.2219 | 0.2012 |  |
|  | 2.5394 | 0.2130 | 0.2275 | 0.2139 |  |
|  | 2.4734 | 0.2266 | 0.2498 | 0.2279 |  |
|  | **Overall** | 0.1956 | 0.2310 | 0.1974 |  |

# Rxpct factors in resolution bins at cycle 3


|  |  |  |  |  |  |  |  |  |
| --- | --- | --- | --- | --- | --- | --- | --- | --- |
|  | **<d> (Å)**  Rxpct\_Work | Rxpct\_Free | Rxpct\_All | |  |  |  |  |
|  | 15.4464 | 0.3508 | 0.4553 | 0.3548 |  |
|  | 8.9180 | 0.2294 | 0.2531 | 0.2308 |  |
|  | 6.9078 | 0.2194 | 0.2558 | 0.2215 |  |
|  | 5.8382 | 0.2194 | 0.2974 | 0.2220 |  |
|  | 5.1488 | 0.1781 | 0.2116 | 0.1799 |  |
|  | 4.6573 | 0.1584 | 0.2002 | 0.1607 |  |
|  | 4.2841 | 0.1631 | 0.1922 | 0.1647 |  |
|  | 3.9882 | 0.1612 | 0.2202 | 0.1639 |  |
|  | 3.7463 | 0.1678 | 0.2309 | 0.1707 |  |
|  | 3.5436 | 0.1702 | 0.2108 | 0.1724 |  |
|  | 3.3707 | 0.1751 | 0.2224 | 0.1777 |  |
|  | 3.2208 | 0.1824 | 0.2216 | 0.1845 |  |
|  | 3.0893 | 0.1914 | 0.2296 | 0.1932 |  |
|  | 2.9727 | 0.1929 | 0.2025 | 0.1932 |  |
|  | 2.8683 | 0.1961 | 0.1987 | 0.1963 |  |
|  | 2.7743 | 0.1965 | 0.2385 | 0.1984 |  |
|  | 2.6889 | 0.1923 | 0.2027 | 0.1928 |  |
|  | 2.6109 | 0.2018 | 0.2263 | 0.2032 |  |
|  | 2.5394 | 0.2129 | 0.2258 | 0.2137 |  |
|  | 2.4734 | 0.2256 | 0.2435 | 0.2266 |  |
|  | **Overall** | 0.1924 | 0.2300 | 0.1943 |  |
